# The Contribution of Massively Parallel Sequencing in Disclosing Unusual Tri- and Tetra-Allelic Type Patterns Detected at the Multicopy Loci DYS385a/b and DYF387S1

**DOI:** 10.3390/ijms27052434

**Published:** 2026-03-06

**Authors:** Chiara Saccardo, Domenico De Leo, Stefania Turrina

**Affiliations:** Department of Diagnostics and Public Health, Section of Forensic Medicine, University of Verona, P.le L.A. Scuro 10, 37134 Verona, Italy; domenico.deleo@univr.it (D.D.L.); stefania.turrina@univr.it (S.T.)

**Keywords:** forensic genetics, multicopy Y-short tandem repeat, DYS385a/b, DYF387S1, tri-allelic pattern, tetra-allelic pattern, isoallele, massively parallel sequencing

## Abstract

Atypical allelic patterns with additional alleles at multicopy Y-short tandem repeats (Y-STRs), such as DYS385a/b and DYF387S1, mainly arise from duplication or gene conversion events occurring in the palindromic regions of the Y chromosome where these markers are located. Although rarely encountered in forensic genetics, these allelic patterns require accurate deconvolution of the single allelic components to ensure correct genotype interpretation. Capillary electrophoresis (CE), the standard method for STR typing, infers multiallelic Y-STR genotypes through intra-locus and intra-color peak height ratios. However, this approach may be insufficient when the pattern includes isoalleles (alleles identical in length but differing in sequence), potentially leading to an underestimation of the number of alleles and therefore the true allelic configuration. Massively parallel sequencing (MPS) technique, through amplicon sequence-based analysis, provides additional information that can overcome ambiguities and interpretative errors arising from CE analysis of complex Y-STR patterns. In this study, analysis of raw MPS sequence data enabled the identification, in two male-derived DNA samples, of complex tri-allelic patterns at DYS385a/b and DYF387S1 loci, classifiable as type 2-B and 2-C, respectively. Furthermore, in a third male-derived DNA sample, a previously undescribed tetra-allelic pattern was detected at DYF387S1, characterized by an isoallele with double read counts.

## 1. Introduction

The short tandem repeats (STRs) loci located on the Y chromosome (Y-STRs), owing to their non-recombining patrilineal inheritance, have assumed particular investigative relevance in the forensic genetic field when male subjects are involved.

Y-STRs analysis plays a pivotal role in identifying and differentiating male DNA contributors within mixed DNA samples (such as those encountered in sexual assault cases), in resolving paternity (especially deficient paternity cases where the alleged father is unavailable) and kinship tests. Moreover, Y-STRs are applied in familial searching strategies aimed at identifying male-lineage relatives in criminal investigations [1,2,3,4].

Given the haploid nature of the Y chromosome, most Y-STR loci, when analyzed by capillary electrophoresis (CE), typically show a single allelic peak in the electropherogram. Multicopy Y-STR loci, such as DYS385a/b and DYF387S1, which usually display two allelic peaks in either homozygous or heterozygous configurations, represent an exception.

Nevertheless, these loci are located within the non-recombining region of the Y chromosome (NRY), also known as the male-specific portion of the Y chromosome (MSY), which comprises approximately 95% of the Y chromosome, and includes ampliconic segments characterized by palindromic regions, inverted repeats, and a variety of long tandem arrays [5]. The high identity sequence degree among these ampliconic regions renders them particularly prone to gene conversion and non-allelic homologous recombination (NAHR) [6,7,8] processes that can generate a variety of structural rearrangements, including inversions, segmental deletions, duplications within palindrome arms leading to amplicon copies with the same or different number of repeat units, and duplications accompanied by nucleotide substitutions (mutations) [9,10,11].

Consequently, these rearrangements make Y-STR loci prone to copy-number variations (CNVs) [12,13,14], which can occasionally give rise to unusual bi-allelic, tri-allelic, or even tetra-allelic patterns [15,16,17].

Such Y-STR atypical multiallelic configurations are rare, with a variable frequency not exceeding 5%, strongly correlated with the specific locus and the population group under investigation, and, when accurately detected and correctly interpreted, increase the informativeness provided by the marker, which can uniquely characterize specific Y-chromosome haplotypes and contribute to the resolution of phylogenetically informative substructures within Y-chromosome haplogroups [18,19,20,21,22]. Conversely, failure to recognize or misinterpret such patterns may lead to ambiguous or erroneous conclusions in forensic casework, including misclassification as male–male mixtures or sample contamination, or technical artifacts.

Conventional CE-based DNA typing, which relies exclusively on fragment-length analysis, remains the widely adopted gold standard in routine forensic practice due to its reliability, rapid workflow, and results compatibility with international database formats. However, CE could present pitfalls and limitations when interpreting multiallelic genotypes, especially when these are detected at multicopy Y-STR loci, proving to be insufficient to ensure accurate and unambiguous results.

In CE analysis, the true number of alleles in a multiallelic genotype is deduced based on the peak height ratio (PHR) between the genotype’s sister alleles (intra-locus balance) and among loci’s alleles within the same color line (intra-color balance). In some cases, this approach could underestimate the observed allelic imbalance, incorrectly attributing it to a weak amplification yield of one of the two alleles in the genotype due to a primer-binding site mutation rather than a proper duplication event on the sister allele [23,24].

Moreover, the isoalleles (also known as isometric alleles), which are alleles sharing the same length but with different sequences, remain undetected in CE analysis; this further hinders the accuracy of intra-locus peak interpretation, particularly concealing complex tri- or tetra-allelic patterns [25].

The interpretative limitations of CE in analyzing atypical multiallelic Y-STR genotypes could be overcome by employing massively parallel sequencing (MPS), also known as Next-Generation Sequencing (NGS), which enables simultaneous allele characterization based on both their length and sequence [26,27,28,29].

As CE, MPS provides, through allele count ratios (ACRs), a quantitative assessment of allelic representation within the pattern [30], which, however, integrating with sequence-based analysis (that also detects isoalleles), makes MPS able to offer a more reliable metric for distinguishing structural rearrangement events, such as chromosome duplication, from stochastic amplification effects.

Therefore, MPS could contribute to resolving complex multi-allelic Y-STR profiles by correctly identifying additional allele copies and isoalleles, and consequently reducing false-positive male–male mixture interpretations, and, as reported in some studies [11,20,21,25,31], it could also enhance the accuracy of Y-haplotype databases and strengthen the evidential value of Y-chromosomal analysis in forensic casework.

This study presents a comparative evaluation between CE and MPS regarding their ability to resolve complex and atypical tri- and tetra-allelic patterns observed at the multicopy Y-STR loci DYS385a/b and DYF387S1.

By analyzing raw sequencing data generated by MPS, which enable a detailed read-depth-based identification of copy-number variation and characterization of allele sequence variation, the study demonstrates a substantial improvement in the accurate interpretation of unusual allelic configurations that would otherwise remain ambiguous or undetected using CE-based analyses alone.

## 2. Results and Discussion

Multicopy Y-short tandem repeat (Y-STR) loci, such as DYS385a/b and DYF387S1, typically display one or two distinct peaks in the electropherogram, corresponding to homozygous and heterozygous allele combinations, respectively, even if rare allelic patterns characterized by more than two alleles arranged in atypical heterozygous combinations have already been observed [32,33].

In the present study, during data collection involving the Y-STR markers included in the Yfiler™ Plus PCR Amplification Kit (hereafter referred to as YfP) (Thermo Fisher Scientific, Waltham, MA, USA) [34], capillary electrophoresis (CE) analysis revealed heterozygous genotypes characterized by a pronounced peak height imbalance between the two sister alleles at the DYS385a/b and DYF387S1 loci in 2 out of 152 male individuals originating from Northeast Italy.

Anyway, for all samples, including those exhibiting atypical patterns, the number of alleles detected at each Y-STR locus, encompassing the multicopy loci DYS385a/b and DYF387S1, was consistent with the allelic configurations expected for single-source DNA samples, allowing for confident exclusion of the possibility that the observed atypical allelic configurations could be attributable to accidental contamination or sample mixture.

**Figure 1:**
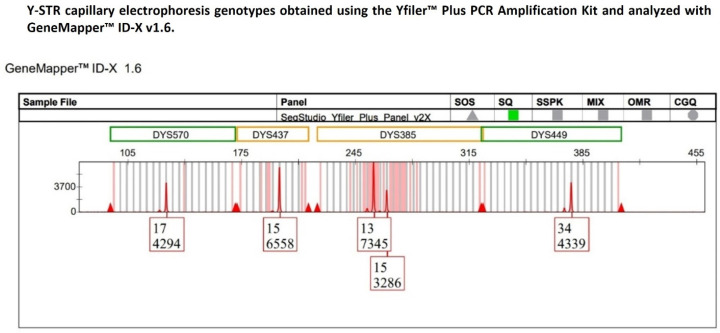
The multicopy DYS385a/b locus displays a heterozygous 13–15 genotype with pronounced peak heights of allelic imbalance, with allele 13 showing a height in RFU approximately twofold that of allele 15, whereas the remaining Y-STR loci within the same dye channel exhibit peak heights consistent with expected analytical variability.

Specifically, in one single-source DNA sample, a heterozygous 13–15 allelic pattern was detected at the DYS385a/b locus, with allele 13 exhibiting a peak height of 7345 relative fluorescence units (RFUs), approximately two-fold higher than that of allele 15 (3286 RFUs) (Figure 1).

For this bi-allelic genotype, the heterozygous balance (HB) (also referred to as the intra-locus balance (ILB)) between the two sister alleles was assessed using the peak height ratio (PHR). The HB value obtained was 0.45, which was substantially below the minimum HB threshold of 0.65 commonly considered indicative of allelic balance in a heterozygous STR genotype analyzed by CE.

To investigate whether the observed imbalance could be attributable solely to amplification-related stochastic effects, the DNA sample was subjected to a second independent amplification using the same YfP kit. Capillary electrophoresis re-typing confirmed the persistence of the imbalance at the DYS385a/b locus to the same extent as initially observed.

Subsequently, allele peak heights of the other Y-STR loci (DYS570, DYS437, and DYS449) detected within the same dye channel as DYS385a/b were compared to determine whether the pronounced imbalance observed at DYS385a/b reflected a locus-specific phenomenon rather than an amplification bias affecting Y-STRs sharing the same fluorescent label.

The choice to restrict the comparison to only those Y-STRs labeled with the same fluorescent dye as the DYS385a/b locus was consistent with previous studies demonstrating that, in capillary electrophoresis-based STR analysis, dye-dependent differences in fluorescence intensity within STR multiplexes can significantly affect inter-locus balance estimation, thereby justifying the evaluation of peak height variability within a single dye channel to minimize potential dye-related bias [35,36].

Furthermore, given the duplicated nature of the DYS385a/b locus, with fragments a and b located on inverted arms within palindromic region 4 (P4) of the long arm of the Y chromosome and separated by an approximately 40 kb unique spacer sequence, the locus was treated as consisting of two independent loci, namely DYS385a and DYS385b, which were therefore evaluated separately [37,38,39].

Accordingly, the intra-color balance (ICB) was evaluated on the five Y-STR loci labeled with the TAZ^TM^ fluorescent dye within the red dye channel of the YfP kit (DYS570, DYS437, DYS449, DYS385a, and DYS385b) using both the coefficient of variation (CV), expressed as a percentage, and the normalized (standardized) z-score. These two statistical metrics provide a quantitative framework to assess the presence of DNA sample-independent amplification bias.

Notably, the coefficient of variation gives the percentage of data variability, expressed as standard deviation, with respect to the mean, which, in this context, refers to the variability in signal intensity related to the RFU distribution among alleles of the Y-STRs within the same dye channel.

The two replicates yielded comparable %CV values of 33.07% and 35.43%, respectively, and although no strict thresholds have been established for %CV interpretation, values within the 25–50% range are generally regarded as indicative of moderate variability in overall signal intensity across the dataset, consistent with expected analytical performance.

However, %CV, providing a global measure of signal variability, did not allow for the identification of which specific Y-STRs deviated from the mean and contributed disproportionately to this variability. Hence, for locus-specific assessment, the normalized z-score analysis was applied, which could represent a more suitable approach, given that it enabled a standardized comparison of peak height deviations for each locus from the expected mean distribution; interpretation based on defined z-score intervals (i.e., values close to zero, ±1, and ±2) would facilitate the identification of outlier loci exhibiting abnormally low or high signal intensities.

**Figure 2:**
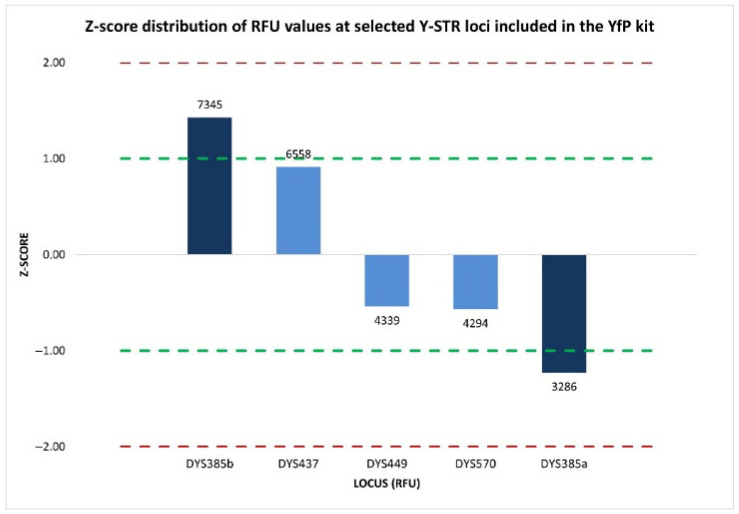
Normalized z-score distribution of relative fluorescence unit (RFU) values of peak heights for Y-STR loci detected within the same dye channel of the YfP kit. The loci show z-score values within the ±1 interval, whereas the two components of the duplicated DYS385a/b locus display opposing deviations from the mean, indicating a locus-specific allelic imbalance. Dashed lines represent the ±1 and ±2 z-score thresholds.

The z-score analysis revealed a heterogeneous distribution of signal intensities among the five Y-STR loci evaluated within the same dye channel. Specifically, the two components of the duplicated DYS385 locus displayed opposing moderate deviations from the mean, where DYS385b showed a positive z-score of 1.428, corresponding to a moderately elevated signal intensity, whereas DYS385a exhibited a negative z-score of −1.230, indicating a moderately reduced signal (Figure 2).

Although neither value exceeds the ±2 threshold typically associated with significant deviations from the expected distribution, the z-score values for the two sister fragments were however not consistent with those obtained for the remaining Y-STR loci (DYS437, DYS449, and DYS570) labeled with the same dye, all of which displayed z-score values within ±1, reflecting signal intensities consistent with the analytical variability generally expected.

**Figure 3:**
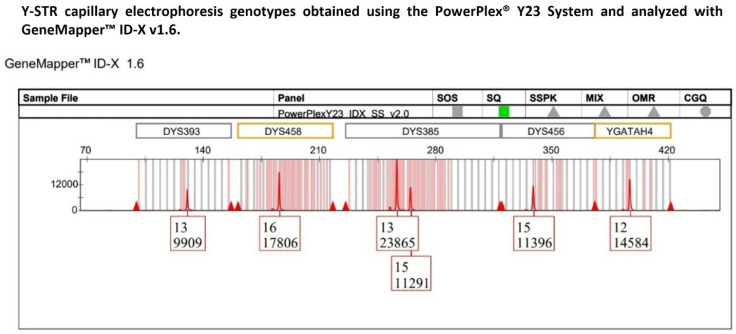
The multicopy DYS385a/b locus displays a heterozygous 13-15 genotype with pronounced peak heights of allelic imbalance, with allele 13 exhibiting a height in RFU approximately twofold that of allele 15, whereas the remaining Y-STR loci detected within the same dye channel show peak heights consistent with expected analytical variability.

Therefore, to rule out the possibility that the observed imbalance was traceable to the YfP kit-specific effects, the same DNA sample was subsequently reanalyzed using the PowerPlex^®^ Y23 System (hereafter referred to as PPY23) amplification kit [40]. Peak height analysis was performed on the confirmed 13-15 heterozygous genotype detected at the DYS385a/b locus (Figure 3) to evaluate the heterozygous balance, and on all the Y-STR loci that, within the PPY23 kit, were labeled with the same fluorescent dye (CXR-ET) as DYS385a/b. Similarly to the previous analysis with the YfP kit, the DYS385a/b locus was treated as comprising two distinct loci to assess the uniformity of peaks’ signal intensities across the dye channel.

The resulting HB and %CV values (0.47 and 35.62%, respectively) were largely consistent with those previously obtained using the YfP kit, confirming the presence of intra-locus imbalance at DYS385a/b and moderate variability in signal intensity distribution across loci.

Subsequent z-score analysis, used to identify which locus in the same dye channel had an abnormal intensity, revealed that the loci DYS393 (−1.02), DYS458 (0.62), DYS456 (−0.71), YGATAH4 (−0.05), and DYS385a (−0.73) exhibited values within or in close proximity to the ±1 interval, indicative of an overall signal intensity distribution that is highly consistent and closely aligned with the mean RFU distribution, reflecting expected analytical variability.

**Figure 4:**
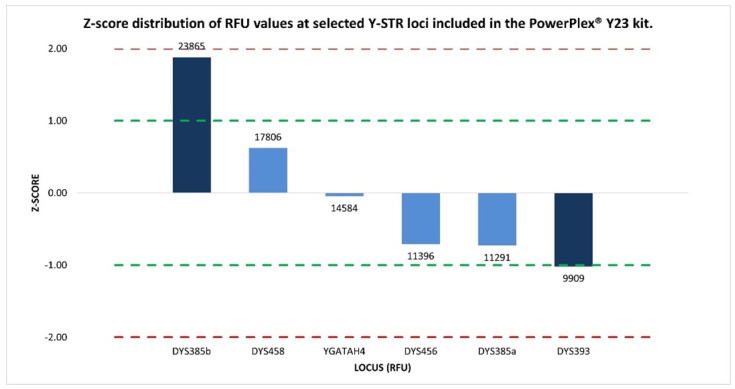
Normalized z-score distribution of relative fluorescence unit (RFU) values’ peak heights for Y-STR loci detected within the same dye channel of the PPY23 kit. The loci show z-score values within the ±1 interval, consistent with expected analytical variability, whereas the duplicated DYS385 locus displays asymmetric deviations, in particular with DYS385b exhibiting a markedly elevated RFU value. Dashed lines indicate the ±1 and ±2 z-score thresholds.

The only exception was DYS385b, which was characterized by a markedly elevated signal intensity (23,865 RFUs), approximately twofold higher than that observed for the other Y-STR loci, and was associated with a z-score of 1.88, representing the most pronounced deviation among the analyzed markers and clearly distinguishing DYS385b from the remaining loci within the same dye channel (Figure 4).

Although the two Y-STR kits have generated profiles characterized by peaks with different heights in absolute RFU values, the persistence of the observed allelic imbalance and the substantial concordance of the investigated quantitative metrics (HB, %CV, and z-score) across independent amplifications indicated a stable and reproducible amplification asymmetry between the two duplicated fragments of DYS385.

This consistency argues against the hypothesis of random stochastic effects arising from dye-channel-related analytical effects or Y-STR multiplex kit-specific influences, suggesting that the observed imbalance may be associated with a locus-specific peculiarity of the analyzed template DNA sample. This would turn out consistent with the genomic structure of the DYS385a/b locus, whose duplicated fragments, DYS385a and DYS385b, being located within the inverted arms of palindromic region 4 (P4) in the Y chromosome’s long arm, are prone to structural rearrangements or to sequence variations, including primer-binding site mutations, that could differentially affect their amplification efficiency.

Since only targeted sequence-based approaches can explain the interpretative uncertainties left by Y-STR capillary electrophoresis regarding the occurrence of the atypical imbalanced bi-allelic pattern, all male DNA samples were subsequently analyzed by massively parallel sequencing (MPS) on the MiSeq FGx™ Sequencing System (Illumina, San Diego, CA, USA) [41], employing the DNA Primer Mix A (DPMA) included in the ForenSeq™ DNA Signature Prep Kit (Verogen, San Diego, CA, USA) (hereafter referred to as FSSP) [42].

Genotyping results derived from sequencing data generated by the Universal Analysis Software v1.2 (hereafter referred to as UAS) [43] on the MiSeq FGx platform were fully concordant with those obtained by CE using the YfP and PPY23 kits for all Y-STR loci across the 125 DNA samples analyzed.

**Figure 5:**
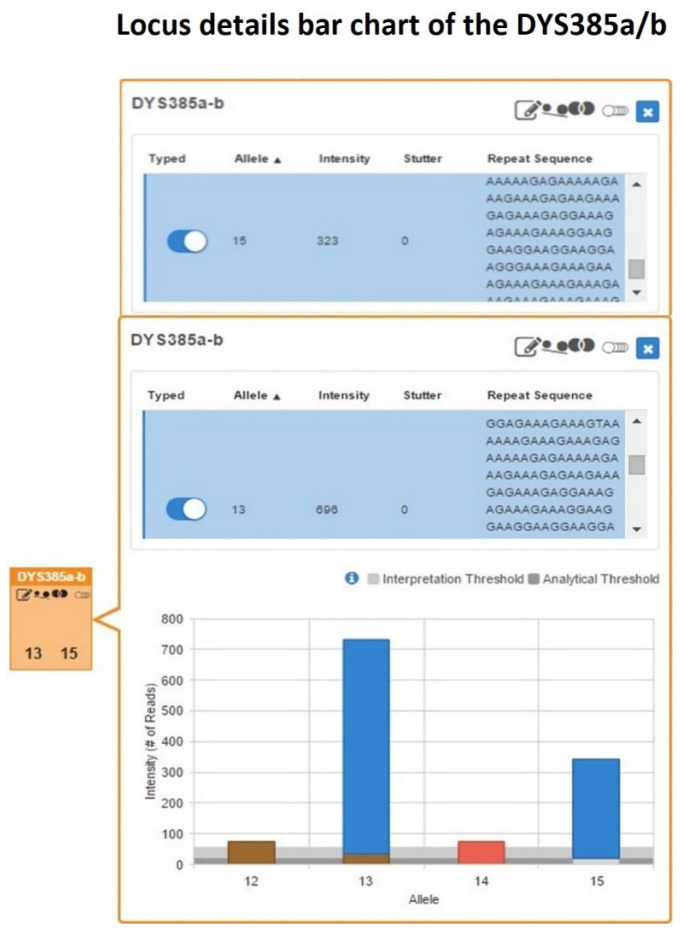
DYS385a/b locus details bar chart showing a heterozygous 13-15 genotype with pronounced read count imbalance, where allele 13 is supported by approximately twice the read depth (696 reads) compared with allele 15 (323 reads).

Specifically, in the UAS Sample Details Table for the single-source DNA sample, the allele count and allelic imbalance quality control icons were automatically flagged for the genotype 13-15 at the DYS385a/b locus (Figure 5).

Subsequent inspection of the Y-STR Excel worksheet in the Sample Genotype Report confirmed the presence of a pronounced imbalance within this heterozygous configuration, with allele 13 supported by approximately twice the number of reads (696 reads) compared with allele 15 (323 reads).

The intra-locus balance between the two sister alleles, evaluated as the allele count ratio (ACR), yielded a value of 0.46, which was below the minimum heterozygous balance (HB) threshold of 0.60 commonly adopted to indicate allelic balance in MPS-based STR interpretation, thus confirming the pronounced imbalance in the heterozygous pattern already identified by Y-STR CE analysis.

Also in this context, to rule out that the observed allelic imbalance was driven by stochastic effects inherent to the sequencing assay employed, inter-locus balance in sequence read coverage was assessed using both %CV and the normalized z-score. As suggested by previous studies [44,45], to minimize heterogeneity of read depth that can arise when comparing markers of different classes, the analysis was performed exclusively on markers of the same type included in the DPMA panel of the FSSP kit. However, since the focus of the present study was on multicopy Y-STRs, in order to ensure a more meaningful interpretation of inter-locus read variability, the comparisons were further restricted to multiallelic loci only, namely the 27 autosomal STRs and the two multicopy Y-STR loci DYS385a/b and DYF387S1 included in DPMA.

The %CV value obtained was 68.14%, falling within the 50–75% range, reflecting a substantial dispersion in read coverage across the analyzed loci; nevertheless, this level of variability is considered intrinsic to MPS-based STR analysis and does not necessarily imply that the heterozygous imbalance observed at DYS385a/b is a direct consequence. Indeed, as reported in previous studies [44,45], in MPS-based analyses, an inhomogeneity in locus-specific sequencing depth may be considered as an inherent aspect of the methodology, given the multitude of factors influencing amplification and sequencing efficiency [46].

**Figure 6:**
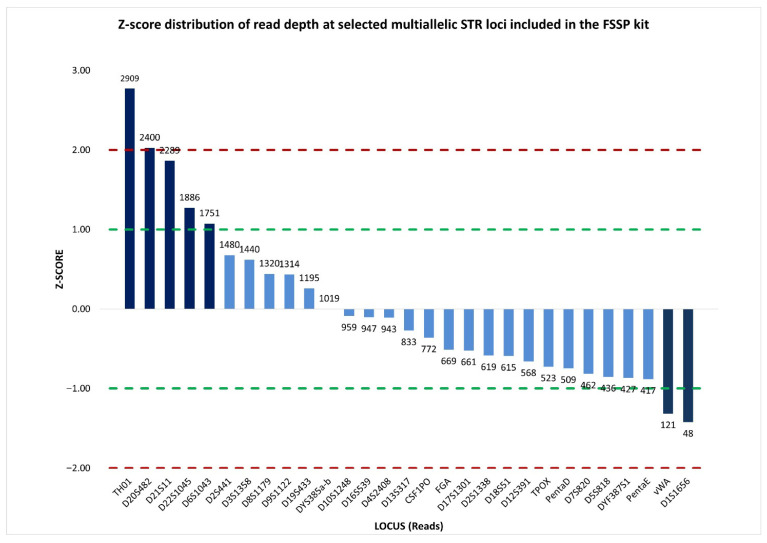
Normalized z-score distribution of sequence read counts for selected multiallelic STR loci analyzed by massively parallel sequencing using the ForenSeq™ DNA Signature Prep (FSSP) kit. Most STR loci display z-score values within the ±1 interval, including DYS385a/b, indicating read coverage consistent with expected sequencing variability, whereas a limited subset of loci exhibits pronounced positive or negative deviations. Dashed lines indicate the ±1 and ±2 z-score thresholds used to identify loci with abnormally high or low read depth.

Therefore, a more informative representation of sequence read depth was provided by the standardized locus-by-locus comparison of read coverage, which revealed a substantial concordance in read distribution across loci. Specifically, 22 out of 29 multiallelic STRs, including DYS385a/b, showed read counts close to the mean, with z-scores falling within the ±1 interval. This indicated that read coverage at DYS385a/b was consistent with that of the other loci, and supported the interpretation that the observed imbalance was unlikely to be attributable to stochastic sequencing effects (Figure 6).

These findings corroborated the capillary electrophoresis results, supporting that a stable locus-specific amplification asymmetry between the two alleles at DYS385a/b was consistently observed, independent of the analytical technique or kit employed. Consequently, the allelic imbalance observed at DYS385a/b could plausibly arise from only two alternative events: (i) sequence mutations affecting the locus-specific primer-binding sites, leading to a reduced amplification efficiency of one of the two amplicons during the PCR-based library preparation [47,48]; or (ii) a copy-number variation in the target fragment, arising from duplication of the segment located on one of the two inverted arms of palindrome 4, resulting in a twofold amplification of one of the two amplicons.

Generally, to exclude the presence of locus-specific primer-binding site mutations, extended Sanger sequencing of the flanking regions surrounding the repeat sequence is performed; in the present study, this assessment was instead attempted by exploiting the complete raw sequences generated by MPS.

From the initial comparison of the UAS-processed sequence strings reported in the Excel Sample Details Report for the DYS385 fragments a and b with the corresponding reference sequences from the Forensic Sequence STRucture Guide v6.1 beta (hereafter referred to as FSSG) [49,50,51] revealed no sequence variation within the repeat core and within the immediately adjacent upstream and downstream flanking regions, although evaluation of the these latter regions was limited to 12 upstream and 119 downstream nucleotides, respectively (Table S1). This finding suggests that the observed allelic imbalance was unlikely to originate from sequence variation within the repeat region or its proximal flanking sequences.

A more extensive sequence comparison was made possible by using the sequence strings of the two DYS385a/b fragments generated by STRait Razor v3 (hereafter referred to as SRv3) under its default configuration (ForenSeqv1.27.config) [52]. This pipeline analyzes the raw sequences reported in the forward Read 1 (R1) FASTQ files, which, having not yet undergone UAS trimming and retaining a length of 351 nucleotides each, allowed us to extend the comparison with the FSSG reference sequences, after adjusting their strand orientation to align with FSSG, to 142 nucleotides in the reverse flanking region of DYS385b and the complementary forward flanking region of DYS385a, without detecting any sequence variation (Table S1). However, for one flanking region, corresponding to the forward flanking region of DYS385b and the complementary reverse flanking region of DYS385a, both bioinformatic tools restricted the evaluation to a sequence span of only 12 nucleotides, which is too short in length to rule out mutations at locus-specific primer-binding sites effectively.

To maximize coverage of the flanking regions, also in accordance with ISFG recommendations for MPS-based STR analysis, which advise extending examination to at least 100 nucleotides adjacent to each side of the repeat core [49,50], sequence analysis was extended to the full 351-nucleotide sequence strings reported in the sample-specific FASTQ file.

To facilitate manual inspection and reduce file size and complexity, the sample’s R1 FASTQ file was converted to FASTA format using the Galaxy online platform [53], and the sequence strings were subsequently extracted and transferred into an Excel worksheet. Using the repeat cores corresponding to alleles 13 and 15 as references, only the raw sequence strings associated with these two alleles were selected for downstream analysis.

Alignment of the selected sequence strings with those reported in the FSSG and with the current human reference genome (GRCh38/hg38), accessible via the UCSC Genome Browser, demonstrated concordance across 42 nucleotides upstream and 148 nucleotides downstream of the repeat motifs (Table S1). Notably, no sequence variation was detected within the 42-nucleotide overlapping region between the forward flanking sequence of DYS385b allele 13 and the complementary reverse flanking sequence of DYS385a allele 15. This concordance further supports the exclusion of sequence-level variation in the analyzed flanking regions as a contributing factor to the observed allelic imbalance at the DYS385a/b locus.

Moreover, within the 42-nucleotide overlapping portion between the forward flanking sequence of DYS385b allele 13 and the complementary reverse flanking sequence of DYS385a allele 15, the forward primer sequence (5′-CCAATTACATAGTCCTCCTTTC-3′) was identified [54,55], and showed complete sequence alignment, mapping in chrY:18,639,683-18,639,704 for fragment b and chrY:18,680,717-18,680,696 for fragment a (Table S1). As no sequence variations were detected at the primer-binding sites within the template DNA of the sample across the two complementary flanking regions, the possibility that the allelic imbalance observed at DYS385a/b was caused by a primer-binding site mutation leading to reduced amplification of allele 15 during PCR-based library preparation could be excluded.

In the absence of evidence supporting a primer-binding site mutation event, it seems strongly supported that a duplication event occurred involving the P4 palindromic arm harboring DYS385b, leading to an additional copy of the DYS385b fragment, resulting in an increased dosage of the DYS385b allele and, consequently, an approximately twofold higher amount of amplicon corresponding to allele 13 compared to allele 15.

Therefore, based on quantitative metrics that were consistent between CE- and MPS-based analyses, combined with a comprehensive inspection of the raw sequence strings generated by MPS, it was possible to establish that the imbalanced bi-allelic pattern observed at the DYS385a/b locus actually represented an unusual tri-allelic configuration, consisting of two identical copies corresponding to allele 13 and one copy corresponding to allele 15. According to the tri-allelic pattern classification framework originally proposed by Clayton [56], which distinguishes between Type 1 and Type 2 patterns based on the mechanism generating the additional allele, the tri-allelic pattern herein corresponds to Type 2, arising from structural rearrangement. However, a further refinement of this type of pattern can be achieved by applying the classification proposed by Picanço et al. [57], which, based on both quantitative balance and allelic configuration, subdivides Type 2 tri-allelic patterns into Types 2-A, 2-B, and 2-C.

Within this classification, Type 2-B is characterized by the presence of a double-dose signal, referring to two identical allelic copies, associated with a single-dose signal, referring to another different allelic copy; this configuration matches the tri-allelic pattern herein observed at the DYS385a/b locus.

A second heterozygous genotype showing an extremely pronounced imbalance was identified at the multicopy DYF387S1 locus in the genetic profile obtained using the YfP kit from a single-source DNA sample.

**Figure 7:**
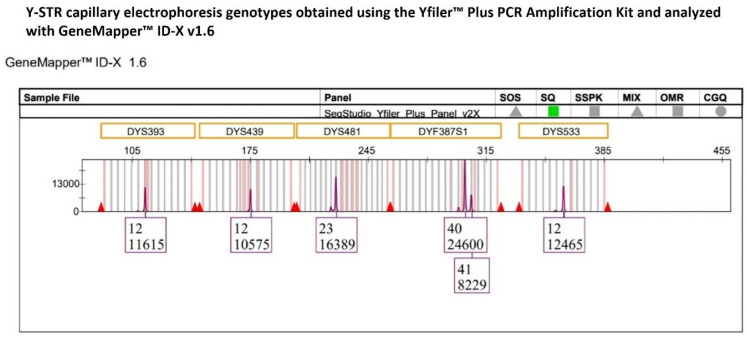
The multicopy DYF387S1 locus displays a heterozygous 40-41 genotype with marked peak heights of allelic imbalance, with allele 40 showing a height in RFU approximately threefold that of allele 41, whereas the remaining Y-STR loci detected within the same dye channel exhibit peak heights consistent with expected analytical variability.

In the electropherogram, the genotype was characterized by an allele 40 with a peak height approximately threefold (24,600 RFUs) the allele 41 (8229 RFUs) (Figure 7), resulting in an HB value of 0.33, which is well below the commonly accepted minimum threshold of 0.65. This finding was consistent with an approximate 3:1 allelic ratio, suggestive of the presence of a tetra-allelic configuration at the DYF387S1 locus. This pronounced imbalance was subsequently confirmed following a second, independent amplification of the same single-source DNA sample.

To assess whether this observed imbalance could be attributed to amplification-related artefacts or stochastic effects associated with the YfP kit, the intra-color peak height consistency was evaluated among Y-STR loci labeled with the same fluorochrome channel.

Given that DYF387S1 fragment 1 and fragment 2 are located on two inverted arms within the palindromic 1 (P1) region of the long arm of the Y chromosome and are separated by an approximately 2.1 Mb unique spacer sequence, the two fragments were considered as independent loci and evaluated separately.

**Figure 8:**
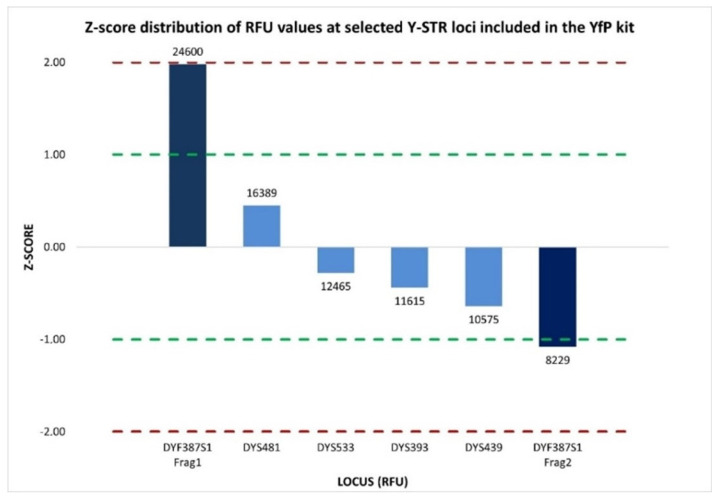
Normalized z-score distribution of relative fluorescence unit (RFU) peak heights for Y-STR loci detected within the same dye channel of the YfP kit. Most loci fall within the ±1 interval, whereas the two DYF387S1 fragments exhibit asymmetric deviations, with fragment 1 showing a marked positive z-score and fragment 2 showing a negative one, consistent with a locus-specific imbalance rather than a dye-related or multiplex-wide amplification effect. Dashed lines indicate the ±1 and ±2 z-score thresholds.

Accordingly, the intra-color balance assessment was extended to six Y-STR loci (DYS393, DYS439, DYS481, DYF387S1 fragment 1, DYF387S1 fragment 2, and DYS533) labeled with the SID™ dye within the purple dye channel. The %CV values calculated from the Y-STRs’ peak height within the electropherograms of two amplification replicates were 41.85% and 56.76%, respectively, indicating medium-to-high variability in overall signal intensity across the loci set. However, locus-specific evaluation using normalized z-score value ascertained that five of the six Y-STR loci (DYS393, DYS439, DYS481, DYF387S1 fragment 2, and DYS533) exhibited homogeneous signal intensity distributions that were closely aligned with the mean. The only exception was DYF387S1 fragment 1, which exhibited a z-score of +1.98, approaching the +2 threshold, indicative of a markedly higher RFU distribution than the mean of the other Y-STR loci (Figure 8). This indicates that the observed medium-to-high %CV values were predominantly driven by increased signal intensity at a single locus, the DYF387S1 fragment 1, rather than by a widespread inter-locus signal dispersion.

The overall evaluation of these results supports the interpretation that the pronounced allelic imbalance detected by CE at the DYF387S1 locus can reflect a genuine tetra-allelic pattern, most likely arising from the presence of three copies of allele 40 and a single copy of allele 41.

Nevertheless, since CE analysis is limited only to fragment-length determination, it does not provide any information on the nucleotide sequences of DYF387S1 fragments 1 and 2, precluding a more detailed characterization of the allelic configurations in the genotype and, in particular, prevents the assessment of whether the observed triple dosage of allele 40 could be attributed, for example, to the presence of isoalleles, namely alleles sharing the same length but differing in their nucleotide sequence, thus necessitating an analysis approach based not only on the length but also on the sequence of the fragments.

Considering that all 152 male DNA samples had been analyzed using the DPMA of the FSSP kit, MPS data were also available for the single-source DNA sample in which CE had detected a marked allelic imbalance at the DYF387S1.

**Figure 9:**
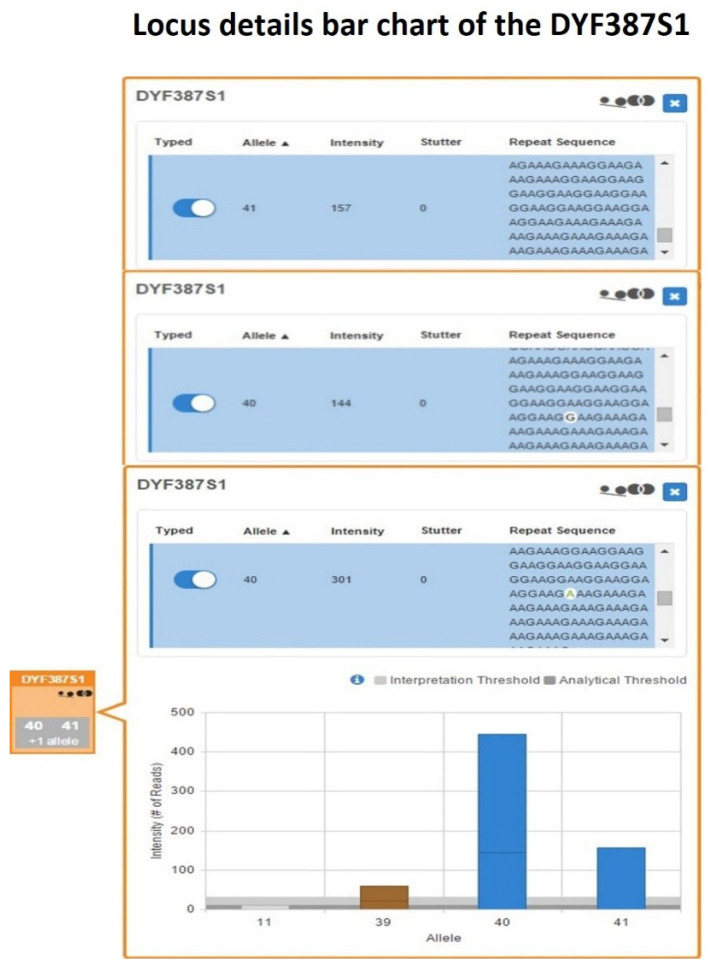
DYF387S1 locus details bar chart showing a heterozygous 40-41 genotype, in which the bar corresponding to allele 40 contains a horizontal demarcation line indicating the presence of two distinct sequences differing by a single-nucleotide variant, highlighted in white within the blue sequence rows.

Length-based genotyping performed using UAS and further reviewed with SRv3 confirmed the DYF387S1 genotype previously assigned by CE as 40-41. However, within the DYF387S1 box of the UAS’s Sample Details Table, the quality flag icons for allele count and allelic imbalance were activated. From the inspection of the corresponding bar graph, a horizontal line in the single bar for allele 40 was present, delineating two different sequences characterized by a single-nucleotide variant (Figure 9).

When the inspection moved to the Y-STRs Excel worksheet in the UAS Sample Genotype Report, the presence of two sequence strings corresponding to allele 40 was revealed, identical in length but differing in their nucleotide composition.

After the alignment of the two UAS-processed allele 40 sequence strings with the reference sequence reported in GRCh38/hg38, a single-nucleotide substitution (A>G) within the repeat region located at the position chrY:25,884,632 was identified, corresponding to SNP rs369894522 (Table S2).

This result demonstrated the presence of a pair of isoalleles for allele 40, herein designated as 40-G and 40-A. To further investigate the presence of additional sequence variations not detectable by the two bioinformatic pipelines employed, a manual inspection of the full raw sequence strings contained in the sample-specific R1 FASTQ file was performed for DYF387S1 fragments 1 and 2 through alignment with the corresponding reference sequences reported in both the FSSG and GRCh38/hg38 assemblies. No additional sequence variations were identified beyond the previously detected SNP, either within the 28 and 53 nucleotide flanking regions surrounding the repeat sequence or within the forward and reverse primer-binding sites, whose annealing positions on the template DNA had been mapped (Table S2) [20,58,59].

To exclude the possibility that the observed imbalance resulted from PCR-dependent stochastic effects, inter-locus balance in sequence read coverage among multiallelic STRs (27 autosomal STRs and two multicopy Y-STRs) was assessed using both the percentage coefficient of variation and normalized z-score metrics.

**Figure 10:**
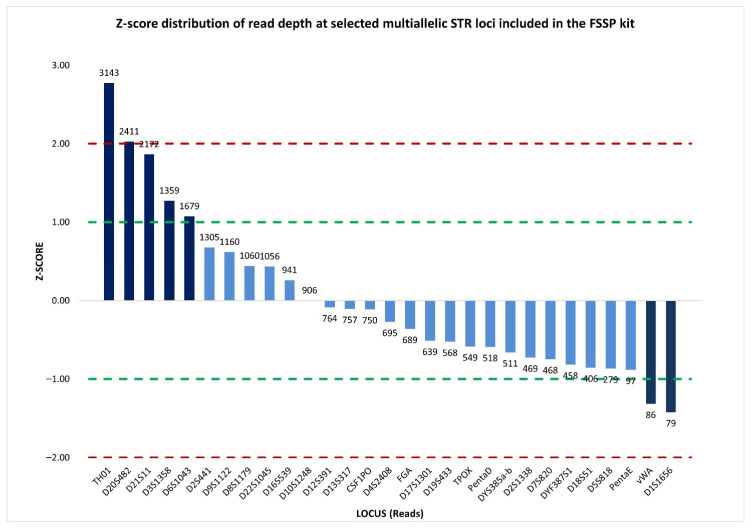
Normalized z-score distribution of sequence read counts for selected multiallelic STR loci analyzed by massively parallel sequencing using the FSSP kit. The majority of loci exhibit z-score values within the ±1 interval, including DYF387S1, consistent with expected sequencing variability, whereas a limited subset of loci shows pronounced deviations. This distribution indicates that inter-locus variability is driven by specific loci rather than by a generalized sequencing imbalance. Dashed lines represent the ±1 and ±2 z-score thresholds.

The %CV value of 78.83% reflected substantial dispersion in read coverage across the analyzed loci; however, normalized z-score analysis revealed that this elevated %CV was driven by a limited number of autosomal loci with z-score values exceeding ±1, which were characterized by read counts markedly higher or lower than the mean. In contrast, 76% of the loci, including DYF387S1, showed read coverage distributions close to the mean, with normalized z-score values within the ±1 interval (Figure 10), indicating that the imbalance at DYF387S1 was unlikely to be attributable to stochastic sequencing variability.

Evaluation of intra-locus balance based on comparison of read counts among individual alleles revealed a clear imbalance within the DYF387S1 genotype. The two isoalleles 40-G and 40-A, with read coverages of 144 and 301, respectively, were present in an approximate 1:2 ratio, corresponding to an allele coverage ratio of 0.48, indicating allelic imbalance. Likewise, allele 41, with 157 reads, showed a similar imbalance relative to allele 40-A (ACR = 0.52), with an approximate 1:2 ratio, while, conversely, it exhibited an approximately 1:1 ratio with allele 40-G, corresponding to a balanced relationship (ACR = 0.92).

Therefore, by integrating the MPS-generated sequence data with quantitative read-depth metrics, it was possible to demonstrate, within this single heterozygous genotype, the presence of three copies of allele 40, resulting from a pair of isoalleles (40-G and 40-A) in which one isoallele (40-A) was present in double dosage relative to the other, together with a single copy of allele 41, thereby confirming the presence of a tetra-allelic pattern initially hypothesized based on CE data.

A plausible explanation for the origin of this unusual allelic configuration most likely involves multi-step mutational events affecting the two inverted repeat copies of the DYF387S1 locus located on each arm of palindromic region P1 of the Y chromosome. These events include non-allelic homologous recombination (NAHR) and gene conversion, which, in concert, through duplication processes leading to variation in amplicon copy number and through single-nucleotide substitutions generating microvariant alleles, give rise to the complex tetra-allelic pattern observed [6,7,8,14,21].

**Figure 11:**
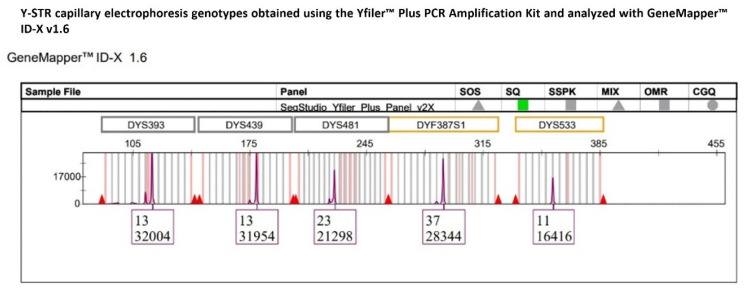
The multicopy DYF387S1 locus was genotyped as homozygous allele 37, with a peak height consistent with those of the other Y-STR loci within the same dye channel, not raising interpretative doubts under CE-based analysis.

**Figure 12:**
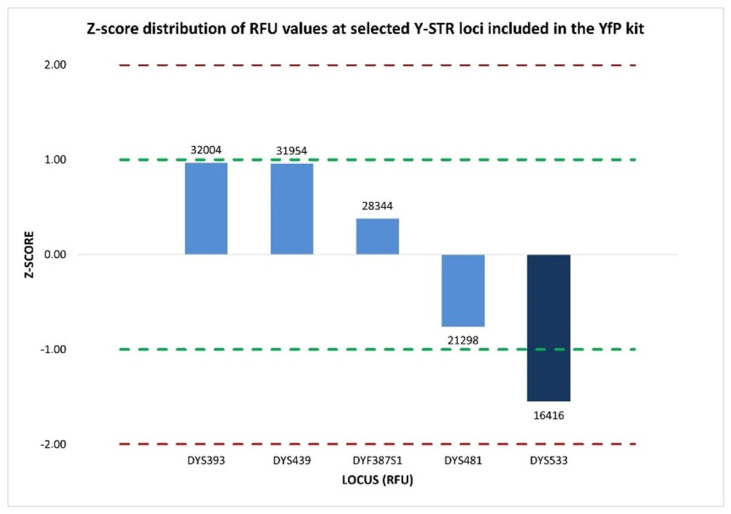
Normalized z-score distribution of relative fluorescent unit (RFU) values of peak heights for Y-STR loci detected within the same dye channel of the YfP kit. With the exception of DYS533, all other loci, including DYF387S1, exhibit z-score values within the ±1 interval, indicating an RFU distribution consistent with expected analytical variability and supporting the CE-based interpretation of a homozygous genotype at DYF387S1. Dashed lines represent the ±1 and ±2 z-score thresholds.

A third unexpected multiallelic pattern at the DYF387S1 locus, which was not detected by YfP-CE genotyping, was identified exclusively in a single-source DNA sample, following an extended inspection of the sequencing data across all 152 Y-STR profiles generated by MPS. Specifically, YfP-CE genotyped DYF387S1 as homozygous for allele 37 (Figure 11), as the corresponding electropherogram peak exhibited a height comparable to those of the other Y-STRs within the same dye channel and did not raise interpretative concerns based on the applied quantitative metrics, which showed %CV values of 26.57% and normalized z-score values close to the mean (Figure 12). These parameters were consistent with an overall signal distribution across loci expected for the applied CE-based analytical approach.

**Figure 13:**
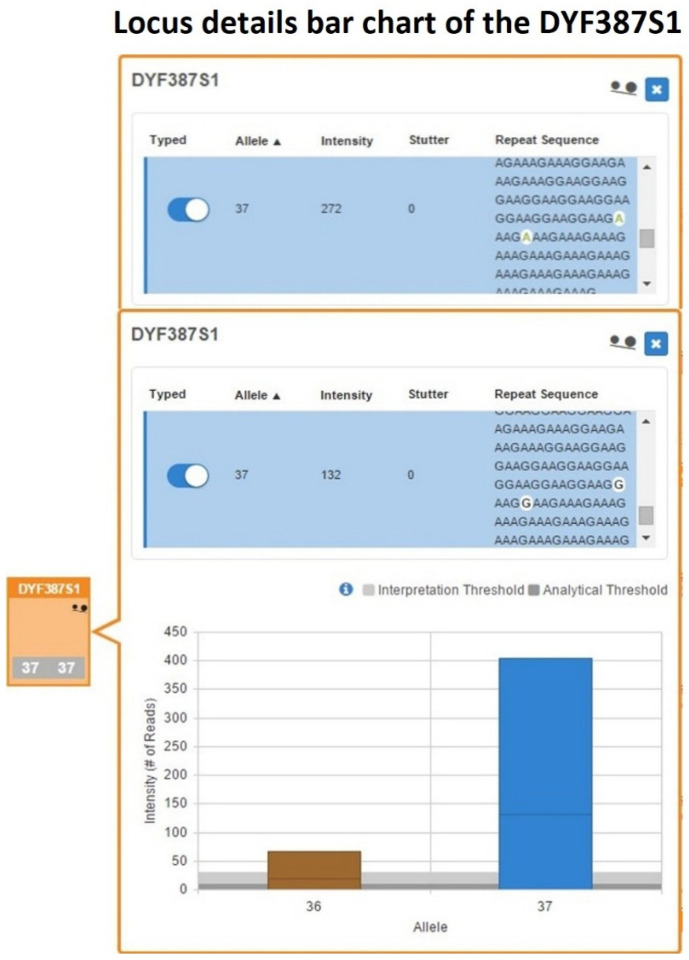
A bar chart detailing the DYF387S1 locus, showing a single bar relative to the allele 37 with a horizontal line delineating two distinct sequences that differ both in nucleotide composition (highlighted in white within the blue sequence rows) and in read count support.

However, in the UAS’s Sample Details Table, within the DYF387S1 box, the quality flag icon indicating allelic imbalance was activated, and in the corresponding bar graph, the single bar for allele 37 showed a horizontal line delineating two different sequences characterized by nucleotide variants (Figure 13).

The inspection of the Y-STRs Excel worksheet within the Sample Genotype Report generated by UAS from the MPS data revealed the presence of two distinct sequence strings for allele 37 that, despite having identical lengths, differed by nucleotide substitutions (A>G) within the repeat region, therefore disclosing the presence of a pair of isoallele 37 variants, herein designated as 37-A and 37-G, whose presence were further confirmed by analyzing of the sequencing data with SRv3.

Subsequent alignment of the raw sequence strings corresponding to DYF387S1 fragments 1 and 2 contained in the sample’s FASTQ file with the reference sequence reported in GRCh38/hg38 confirmed the sequence variations within the repeat strand, allowing for the identification of two nucleotide substitutions at positions chrY:25,884,632, corresponding to SNP rs369894522, and chrY:25,884,628, corresponding to SNP rs2124031964 (Table S3). These substitutions modified the repeat motif structure of the strand, particularly affecting the last two repeat units (A[5]AAGA[2]AAGG[1]TAGG[1]AAGG[3]AAGA[2]AAGG[1]AAGA[2]AAGG[n]AAGA[n]A[3]) [50,60].

No other sequence variations were observed in the forward and reverse flanking regions or in the corresponding primer-binding sites across the two inverted DYF387S1 copies.

**Figure 14:**
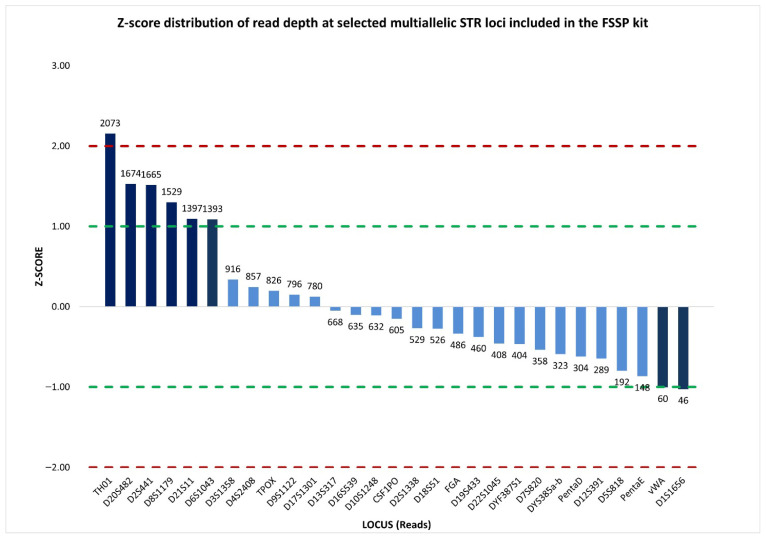
Normalized z-score distribution of sequence read counts for selected multiallelic STR loci analyzed by massively parallel sequencing using the FSSP kit. While a limited number of loci display pronounced positive or negative deviations, the majority of loci, including DYF387S1, show read coverage values within the ±1 interval, indicating that inter-locus variability is driven by specific loci rather than by a generalized sequencing imbalance. Dashed lines indicate the ±1 and ±2 z-score thresholds.

In addition, the assessment of inter-locus balance, based on read depth across multiallelic STR markers (27 autosomal STRs and 2 multicopy Y-STRs), provided a %CV value of 73.17%, reflecting a substantial variability in read coverage among loci. Nevertheless, inspection of the normalized z-score distribution demonstrated that the majority of loci (76%) (Figure 14), including DYF387S1, showed read counts clustering around the mean, suggesting that the elevated %CV was driven by a restricted number of loci with disproportionately low or high coverage rather than by a widespread inter-locus imbalance.

Moreover, the two isoalleles exhibited a marked intra-locus imbalance (ACR = 0.49), where the allele 37-G showed a read count (132 reads) approximately half that of observed for allele 37-A (272 reads), indicating an approximate 1:2 dosage relationship.

Consequently, based on integrative evaluation of MPS-derived sequence data with quantitative read-depth metrics, it was possible to reclassify the genotype initially incorrectly interpreted by CE analysis from a homozygous configuration for allele 37 to a heterozygous configuration exhibiting a tri-allelic pattern due to the combined presence of two identical copies in both length and sequence (37-A) and a single isoallele (37-G).

As observed for the tetra-allelic configurations, the tri-allelic pattern identified at the DYF387S1 locus is plausibly attributable to mutational multi-step events such as non-allelic homologous recombination (NAHR) and gene conversion.

According to the sub-classification of tri-allelic patterns proposed by Picanço et al. [57], the tri-allelic pattern involving allele 37 at DYF387S1 can be classified as Type 2-C, which is characterized by the presence of a single fragment length-based allele occurring in triple dosage.

However, as noted by the authors who proposed this classification, the Type 2-C tri-allelic pattern is extremely difficult, if not impossible, to detect using the CE approach [57], as it typically manifests in the electropherogram as a single peak that does not raise interpretative suspicion about the presence of an underlying tri-allelic configuration. Therefore, in the absence of appreciable peak height discrepancies relative to other STR loci within the same dye channel, the genotype is commonly misinterpreted as a homozygous bi-allelic genotype.

Consequently, it could be that the rarity of the detection of a Type 2-C tri-allelic pattern may be partially attributable to the intrinsic limitations of the PCR-CE-based genotyping technique, which is unable to reveal microvariant or isoallelic sequences contributing to the generation of this tri-allelic configuration.

In contrast, MPS-based analysis readily resolves this level of sequence complexity, enabling the accurate identification and classification of such otherwise cryptic tri-allelic patterns.

## 3. Materials and Methods

### 3.1. DNA Samples, Extraction, and Quantification

In this study, 152 anonymized buccal cell samples obtained from unrelated adult male donors native to Northeast Italy were analyzed after getting their written informed consent and approval from the Research Ethics Committee of the University of Verona (protocol code: CARU-12/2020). Genomic DNA was extracted from each sample using the QIAamp^®^ DNA Mini Kit (Qiagen, Hilden, Germany) in accordance with the manufacturer’s guidelines, and, after quantification performed by Qubit^®^ 2.0 Fluorometer using the Qubit^®^ dsDNA HS Assay Kit (Thermo Fisher Scientific, Waltham, MA, USA), each DNA sample was normalized to a final concentration of 1 ng/μL.

### 3.2. STR Amplification, CE Genotyping, and Data Analysis

All single-source DNA samples were amplified using the Yfiler™ Plus PCR Amplification Kit (YfP) (Thermo Fisher Scientific, Waltham, MA, USA) [34], which comprises 25 Y-STR loci, including the multicopy markers DYS385a/b and DYF387S1 analyzed in this study. Moreover, the positive DNA control (DNA Control 007) supplied with the YfP kit, along with a nuclease-free water sample as a blank control, underwent the amplification process. PCR amplification was carried out according to the manufacturer’s instructions.

Subsequently, Y-STR amplicons were genotyped using the SeqStudio™ Genetic Analyzer for HID (Applied Biosystems, Waltham, MA, USA) [61], and data analysis was performed with GeneMapper™ ID-X v1.6 (Applied Biosystems, Waltham, MA, USA) [62] using the default filter settings recommended by the manufacturer.

A minimum peak height threshold of 100 relative fluorescence units (RFUs) was applied for allele calling at both single-copy and multicopy loci.

Multicopy Y-STR loci, such as DYS385a/b and DYF387S1, typically display one or two peaks in the electropherogram, corresponding to homozygous and heterozygous allele combinations, respectively.

When a heterozygous genotype was observed at these loci, the heterozygous balance (HB), also referred to as intra-locus balance (ILB), between the two sister alleles was assessed using the peak height ratio (PHR), obtained by dividing the lower peak height by the higher peak height, measured in RFUs. A minimum PHR threshold of 0.65 was applied to evaluate acceptable intra-locus balance.

Furthermore, to assess the Y-STR loci’ peak heights intra-color balance (ICB), in which the DYS385a/b and DYF387S1 are included, respectively, both the coefficient of variation expressed as a percentage (%CV) and the normalized (or standardized) z-score were employed.

The %CV, which provides an overall measure of signal uniformity among peak heights of STRs within the same color channel, was calculated by dividing the standard deviation of peak heights by the mean and then multiplying by 100.

A low %CV value generally indicates good peak balance, while a high %CV value suggests greater peak height variability.

The normalized (or standardized) z-score allows for the estimation at each locus of how much a peak height deviates from the expected mean distribution, making it a marker-specific metric. This parameter was calculated by subtracting the mean value of peak heights of the Y-STRs in the same dye channel from the observed peak height at a single locus, and dividing the result by the standard deviation. Z-score values close to 0, corresponding to the calculated mean, indicate a well-balanced peak height across the STRs. Values within ±1 reflect a slight deviation with a moderate but generally acceptable degree of imbalance. Z-score values between ±2 indicate a more pronounced imbalance among loci, although it remains tolerable, whereas values exceeding ±2 denote a marked imbalance, where the outlier values are typically caused by unusually low or high peak heights compared to the expected mean.

After CE analysis, a heterozygous genotype with a marked allelic imbalance at the DYS385a/b locus was identified in one single-source DNA sample, raising the suspicion that more than two alleles were present. To verify the observed pattern, the sample was reamplified with the PowerPlex^®^ Y23 System (PPY23) (Promega Corporation, Fitchburg, WI, USA) [40], which also encompasses the DYS385a/b locus. PCR amplification was performed following the manufacturer’s protocol, employing 2800M control DNA as a positive control and nuclease-free water as the blank PCR control. CE and data analysis were performed as described above.

Since CE analysis was unable to establish whether the intra-locus imbalances or atypical peak patterns observed at DYS385a/b and DYF387S1 were attributable to stochastic PCR effects or to secondary duplication events affecting one of the allelic copies, all 152 DNA samples were subsequently reanalyzed using MPS technology.

### 3.3. MPS Library Preparation, Sequencing, and Data Analysis

For the sequencing analysis, libraries were prepared using 1 ng of each DNA sample with DNA Primer Mix A (DPMA) from the ForenSeq™ DNA Signature Prep Kit (FSSP) (Verogen, San Diego, CA, USA) [42], which contains primers for the amplification of the amelogenin marker, 27 autosomal STRs, 24 Y-STRs (including DYS385a/b and DYF387S1), 7 X-STRs, and 94 identity-informative SNPs, following the manufacturer’s recommended protocol. The 2800M Control DNA provided with the FSSP kit and nuclease-free water samples were included as the positive control and the blank PCR control, respectively.

Sequencing was performed on the MiSeq FGx™ Sequencing System (Illumina, San Diego, CA, USA) according to the manufacturer’s guidelines [41], and the generated raw sequencing data were processed using Universal Analysis Software (UAS) v1.2 (Illumina, San Diego, CA, USA) with the recommended default analytical settings [43]. The analytical threshold (AT) and interpretation threshold (IT), fixed at 1.5% and 4.5%, respectively, outline the minimum proportion of reads required per locus when coverage falls below 650 reads, ensuring that genotype calls are supported by sufficient depth of coverage. Therefore, applying UAS calling criteria, only loci supported by a minimum read depth of 30 reads were considered, ensuring that all reported alleles meet the minimum criteria for calling reliability and are not excluded from the evaluation.

UAS performed allele calling for each Y-STR locus and identified the presence of isoalleles (i.e., isometric alleles, which are amplicons of the same length but with different sequences) in length-based homozygous or heterozygous genotypes at multicopy Y-STRs. To confirm the findings from UAS and to disclose additional sequence variations within the repeat motifs and flanking regions of Y-STRs not detectable by UAS v1.2, raw output FASTQ files generated by UAS for each sample were reexamined using the bioinformatic tool STRait Razor v3 (SRv3) under its default configuration (ForenSeqv1.27.config) [52].

Concerning the MPS quantitative data analysis, similar to the peak height ratio used in CE to assess heterozygous balance, the allele count ratio (ACR), also referred to as allele coverage ratio, was employed in MPS to evaluate intra-locus balance between two sister alleles in each heterozygote and/or isometric genotype detected at DYS385a/b and DYF387S1.

The ACR value was determined by dividing the read count of the lower-coverage allele by that of the higher-coverage allele. A minimum ACR threshold of 0.60, which is the default allele balance threshold of UAS, was used as an indicator of allelic balance, whereas significantly lower ACR values were interpreted as expression of unequal amplification of fragments during library preparation or as potential copy-number variation at the locus.

The inter-locus balance was used to assess whether each multiallelic STR locus within the DPMA panel (represented by 27 autosomal-STRs and 2 multicopy Y-STRs) exhibited a comparable sequence read coverage within the same sample, thereby ensuring that no locus was disproportionately over- or under-represented due to library preparation bias or sequencing variability. Inter-locus balance was estimated using the %CV and z-score normalized obtained from the actual locus-specific read counts after removing reads attributed to noise and stutter artifacts.

Since all forward Read 1 (R1) FASTQ files (i.e., the text-based formats that encode individual sequencing reads and their associated Phred quality scores using a four-line record structure) contain 351 base pair (bp) sequences, which are considerably longer than the sequence fragments reported in the UAS Sample Details Reports for each sample, their analysis enables an extended comparison with the corresponding reference sequences. R1 FASTQ files were converted to FASTA format using the Galaxy web platform [49] and subsequently exported into individual internal Excel worksheets for downstream inspection.

Full allele-specific sequence strings, corresponding exclusively to the DYS385a/b and DYF387S1 loci genotyped in each of the 152 samples, were manually aligned to the reference sequences reported in the Forensic Sequence STRucture Guide (FSSG) v6.1 beta [49,50,51], thus allowing for checking strand orientation and identifying any exceeding sequence portions not included in the reference allele ones. The further check was carried out by sequence alignment to the current human reference genome (GRCh38/hg38) available in the UCSC Genome Browser resources.

The absence of sequence variation in the flanking sequence portion spanning from the Y-STR repeat motifs, in which are also included the primer sequences, may contribute to ruling out the exclusion that unusual allelic configurations, inconsistent with the expected homozygous or heterozygous bi-allelic patterns, arise from primer-binding site mutations affecting amplification and sequencing of the Y-STR amplicons.

## 4. Conclusions

Gene conversion and non-allelic homologous recombination events occurring in palindromic regions of the Y chromosome can generate complex allelic patterns, including tri- and tetra-allelic configurations at multicopy Y-STR loci such as DYS385a/b and DYF387S1. Although heterozygous genotypes are expected at these loci, pronounced peak height imbalances between sister alleles detected by CE are rarely observed in forensic casework and population studies. When present, such imbalances may reflect underlying sequence-level differences, locus-specific amplification dynamics, or structural features of the repeat regions, and therefore warrant further investigation to ensure correct genotype interpretation and to exclude technical artefacts or primer-binding site variations that may affect amplification efficiency.

In this context, the results of the present study demonstrate that MPS plays a crucial role in accurately disclosing hidden alleles and isoalleles that would otherwise remain undetectable by CE-based approaches.

By providing sequence-level resolution, MPS enhances the reliability of Y-STR genotype interpretation and reduces the risk of overlooking unusual tri- or tetra-allelic patterns, which, due to their rarity, contribute to strengthening individual identification capability.

Although conventional CE-based STR analysis remains the gold standard for forensic DNA profiling, the integration of MPS into standard forensic workflows could offer a valuable improvement in the resolution, reliability, and interpretative robustness of complex or unexpected Y-STR profiles.

The combined availability of quantitative and qualitative sequence-based parameters, such as allele coverage and full amplicon sequence strings, allows genuine structural variants to be distinguished from analytical artefacts, ensuring that tri- or tetra-allelic patterns are interpreted as genuine genomic events rather than experimental anomalies. This additional level of resolution, representing a substantial advancement in forensic genetic analysis, supports more robust and defensible interpretations, particularly in judicial contexts.

Overall, while MPS cannot currently be considered an alternative or a replacement for CE due to its ongoing validation, MPS represents a powerful complementary tool that, in selected cases, can overcome specific interpretative limitations of CE and significantly strengthen the analytical framework of Y-STR typing.

## Data Availability

The original contributions presented in this study are included in the article/Supplementary Materials. Further inquiries can be directed to the corresponding author.

## Supplementary Materials

The following supporting information can be downloaded at: https://www.mdpi.com/article/10.3390/ijms27052434/s1.
